# Assessment of dry-fogged hydrogen peroxide as an “untact” room disinfection automation system for rapid terminal decontamination of a single isolation room in a healthcare institution

**DOI:** 10.1186/s13756-025-01613-7

**Published:** 2025-07-26

**Authors:** Jin Woong Suh, Jeong Yeon Kim, Jang Wook Sohn, Sung Eun Lee, Hyeon Jeong Kim, Woo Jin Chi, Mi Na Lee, Young Kyung Yoon

**Affiliations:** 1 Division of Infectious Diseases, Department of Internal Medicine, Korea University Anam Hospital, Korea University College of Medicine, 73, Goryeodae-ro, Seoul, 02841 Republic of Korea; 2 Institute of Emerging Infectious Diseases, Korea University, Seoul, Republic of Korea; 3 Infection Control Unit, Korea University Anam Hospital, Seoul, Republic of Korea; 4 Division of Infectious Diseases, Department of Internal Medicine, Kyung Hee University College of Medicine, Kyung Hee University Hospital at Gangdong, Seoul, Republic of Korea

**Keywords:** Hydrogen peroxide, Decontamination, Infection control, Multidrug resistant, Disinfection

## Abstract

**Background:**

This study evaluates the efficacy of dry-fogged hydrogen peroxide (dHP) as an “untact” room disinfection automation system (URDAS) for rapid terminal room decontamination.

**Methods:**

This prospective study was conducted at a university-affiliated hospital in Korea. After patient discharge, dHP technology was used to decontaminate single rooms. Environmental cultures were collected from inanimate surfaces and room air before and after the decontamination process. Routine manual cleaning and disinfection were performed only after environmental sampling during terminal decontamination.

**Results:**

After applying URDAS using dHP, culture positivity in the surface samples decreased from 20.5% (16/78) to 5.1% (4/78). Particularly, bed-removable tables and bedsheets used by patients often remain contaminated even after disinfection. Thirty-six species were isolated from the air cultures before disinfection, which decreased to 23 species after disinfection, representing a 36.1% reduction. The most frequently isolated pathogens after disinfection were *S. aureus* on fabric materials from surface samples and *Aspergillus* species from air samples.

**Conclusion:**

Our findings demonstrate that URDAS using dHP is an effective tool for disinfecting contaminated environmental surfaces and spaces in single isolation rooms with minimal risk of exposure to medical staff. However, further optimization is required to address the material- and pathogen-specific disinfection challenges.

**Supplementary Information:**

The online version contains supplementary material available at 10.1186/s13756-025-01613-7.

## Background

Despite the ongoing efforts to develop strategies for infection prevention and control, the spread of nosocomial pathogens in hospital environments remains a challenge. Contaminated environmental surfaces in hospital rooms serve as reservoirs of healthcare-associated infections [1, 2]. Particularly, high-touch surfaces near patients are easily contaminated with pathogens shed by infected patients or colonized by multidrug-resistant microorganisms (MDROs). These surfaces can remain contaminated for a few hours or months, and the likelihood of persistent contamination is higher when effective cleaning and disinfection are not performed [3].

Studies have demonstrated that even when environmental cleaning is performed upon patient discharge, less than 50% of room surfaces are adequately disinfected [4, 5]. Even after enhanced environmental disinfection, 5–30% of hospital surfaces remain contaminated [5]. Terminal room disinfection relies on wipe-based methods and is highly operator-dependent, time-consuming, and labor-intensive, leading to increased labor costs. Furthermore, nosocomial pathogens may spread to uncontaminated surfaces during manual cleaning [6]. Terminal disinfection is also associated with technologies such as vaporized hydrogen peroxide (HPV). Notably, disinfection personnel may face exposure risks, particularly during recurrent epidemics of highly pathogenic and contagious infectious diseases.

Airborne microbial particles settle through sedimentation and subsequently contaminate surfaces [7]. Studies have reported a significant correlation between airborne microbial contamination and surface contamination, indicating the need to manage both air and surfaces simultaneously for effective infection control [7, 8].

Autonomous touchless disinfection technologies, such as dry-fogged hydrogen peroxide (dHP), can improve the shortcomings of vaporized hydrogen peroxide, which can easily cause material corrosion due to surface condensation [9, 10], and ultraviolet-C systems, which are less effective in organic-rich environments and leave untreated areas [6]. Technologies such as dHP offer broad-spectrum biocidal efficacy, ease of use, storage stability, and compatibility with various surface materials [10]. The novel disinfection system used in this study is an innovative technology for space disinfection that automates the entire disinfection process and rapidly removes dHP from a room through a catalytic filter and a high-efficiency particulate air filter connected to the heating, ventilation, and air conditioning (HVAC) system. Consequently, user convenience improved, and the running time was dramatically shortened.

However, to the best of our knowledge, no data exist on the assessment of autonomous touchless technologies using dHP for terminal room disinfection in real-world clinical settings. Therefore, this study evaluated the clinical performance of dHP as an “untact” room disinfection automation system (URDAS) for rapid terminal room decontamination.

## Methods

### Study design

**Fig. 1:**
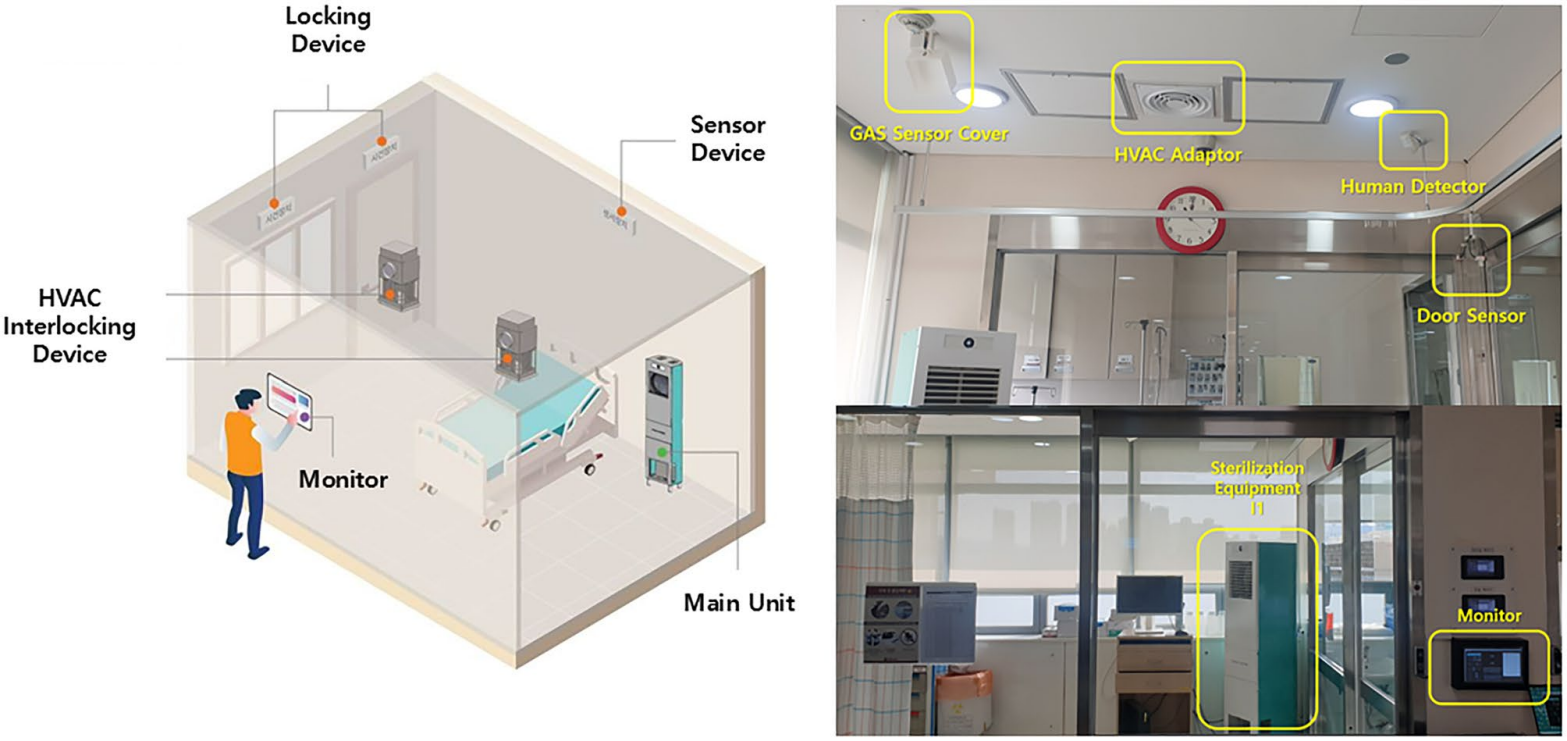
Overall view of an “untact” room disinfection automation system using dry-fogged hydrogen peroxide installed in a single isolation room in the intensive care unit

This prospective study was conducted in single isolation rooms at Korea University Anam Hospital, a 1,048-bed university-affiliated tertiary hospital in Seoul, Republic of Korea. To identify sites contaminated with nosocomial pathogens in single isolation rooms, we conducted environmental swab cultures before terminal room decontamination from January to March 2023. On May 7, 2024, an URDAS using a dHP was installed in a single isolation room in the intensive care unit (Fig. 1). Between May and November 2024, when patients hospitalized for more than 48 h were discharged, URDAS using dHP was applied for terminal room decontamination. To assess the clinical efficacy of dHP as an URDAS for terminal room decontamination, environmental cultures from inanimate surfaces and room air were collected before and after the decontamination process. The study protocol was approved by the Institutional Review Board of Korea University Anam Hospital (approval number: 2022AN0469). All clinical data were obtained using a routine hospital infection control program. Therefore, the requirement for informed consent was waived.

### Definition

Environmental cleaning involves the physical removal of dirt, organic matter, and visible soils from surfaces using detergents or enzymatic cleaners, and is typically performed as a prerequisite for disinfection.

Environmental disinfection involves the application of antimicrobial agents to chemically inactivate pathogens on pre-cleaned surfaces. Terminal room disinfection is a comprehensive process that is conducted after patient discharge to eliminate pathogens from all surfaces, equipment, and air within a room. In our study, the URDAS protocol, as a terminal room disinfection protocol, included routine manual cleaning after the application of dHP disinfection to reduce the risk of exposure of hospital staff performing environmental disinfection.

### Environmental sampling

To evaluate the efficacy of URDAS, environmental sampling was performed twice: immediately after patient discharge and immediately after applying URDAS using dHP. Routine manual cleaning and disinfection were performed only after environmental sampling. This study design simulated an outbreak of potentially lethal infectious diseases in which healthcare staff performing manual cleaning were required to minimize risk exposure. Where possible, the sample sites were standardized to include high-touch surfaces and areas that could have been overlooked during routine environmental cleaning [11]. The Centers for Disease Control and Prevention has provided guidance on best practices for environmental cleaning procedures in healthcare facilities [12]. Preliminary investigations identified the 21 most contaminated locations for environmental sampling (Supplementary Tables S1 and S2). Because of practical constraints, when the surfaces adjacent to a target location were sampled, we assumed a uniform microbial distribution across the entire adjacent surface, as validated in previous studies [8].

Environmental sampling was conducted at 13 sites immediately before and after the application of URDAS using dHP. The sites from which the samples were collected included high-touch surfaces near the patients (bed-removable tables, bedside shelves and rails, bed remote controller, ventilator, infusion lines, infusion pumps, pole stand, and curtains), patient bedding (bedsheets on the head and leg sides), patient monitor, blood pressure cuff, oxygen generator, and suction bottle. A total of 78 samples for surface cultures and 12 samples (six for bacteria and six for fungi) for air cultures were collected before and after disinfection.

To determine whether the reduction in surface bioburden due to URDAS was significant under various conditions, routine manual cleaning and replacement with clean bedsheets were performed only after environmental sampling was completed. Bedsheets were intentionally not removed prior to the application of URDAS using dHP to evaluate the efficacy of the disinfection system on fabric materials, such as bedsheets. These conditions can reduce the exposure of hospital staff to environmental decontamination during an outbreak of a potentially lethal emerging infectious disease. It is also standard practice in Korean hospitals to replace used bedsheets with clean ones during terminal cleaning after patient discharge.

### Microorganism identification and antimicrobial susceptibility tests

All environmental samplings were conducted by the same staff members of the infection control team. Surface cultures were systematically obtained from a 10 × 10 cm^2^ area using a double regular rayon swab (Copan Diagnostics, Murrieta, CA, USA). After the swab tip was placed in sterilized normal saline, the rayon swabs were rotated to ensure full contact between all parts of the swab tip and the surface. In cases where a 10 × 10 cm^2^ surface area could not be obtained, as recommended by the Centers for Disease Control and Prevention guidelines and literature, sterile swabs were used to sample as large an area as possible, adapting the sampling technique to the specific characteristics of each surface [12, 13]. The swab samples were packaged and sent to the microbiological laboratory of our hospital and to an external institution (Seegene Inc., South Korea).

Microorganism identification and antimicrobial susceptibility testing of nosocomial pathogens were performed using matrix-assisted laser desorption/ionization time-of-flight, the semi-automated system MicroScan (Beckman Coulter, Brea, CA, USA), and VITEK II (bioMérieux, Marcy-l’Étoile, France). The laboratory definitions of each MDRO were determined according to the criteria of the Clinical and Laboratory Standards Institute [14].

Air cultures were performed using the Air IDEAL (bioMérieux, Marcy-l’Étoile, France) and DUO SAS SUPER 360 (VWR International PBI, Milan, Italy). For each air culture, 1,000 L of air was sampled. The presence of common bacteria was evaluated using culture media containing tryptic soy agar, incubated at a mean temperature of 36 ± 1 °C for 48 h. Filamentous fungi were detected using plates containing Sabouraud chloramphenicol dextrose agar (SabC, Becton-Dickinson, Heidelberg, Germany), incubated at 30 °C for 10 days, and identified based on macroscopic and microscopic morphological features.

Commercially available *Geobacillus stearothermophilus* (ATCC 12980) bioindicators (Apex Laboratories Inc.; Sanford, NC) were used. These bioindicators consisted of small metal discs coated with 1 × 10^6^ spores and enclosed in Tyvek^®^. Before decontamination, samples were placed at eight sites around the periphery of the unit. The Tyvek^®^-enclosed metal discs attached to each site were aseptically removed immediately prior to manual cleaning after environmental sampling.

### “Untact” room disinfection automation system protocols

The URDAS (STERAPY Co., Ltd., Seoul, Republic of Korea) was designed as an unmanned automated room disinfection system for surface disinfection, surpassing the limitations of traditional wipe-based disinfection methods. The specifications, features, and key functions of URDAS are listed in Supplementary Table S3.

The URDAS was installed in a single isolation room in the intensive care unit. After installation, the engineer provided the infection control staff with training on its use. During the first two operations, the system is operated under the supervision of a technician. Subsequently, the infection control staff operated the system independently and did not complain of any specific difficulties.

**Fig. 2:**
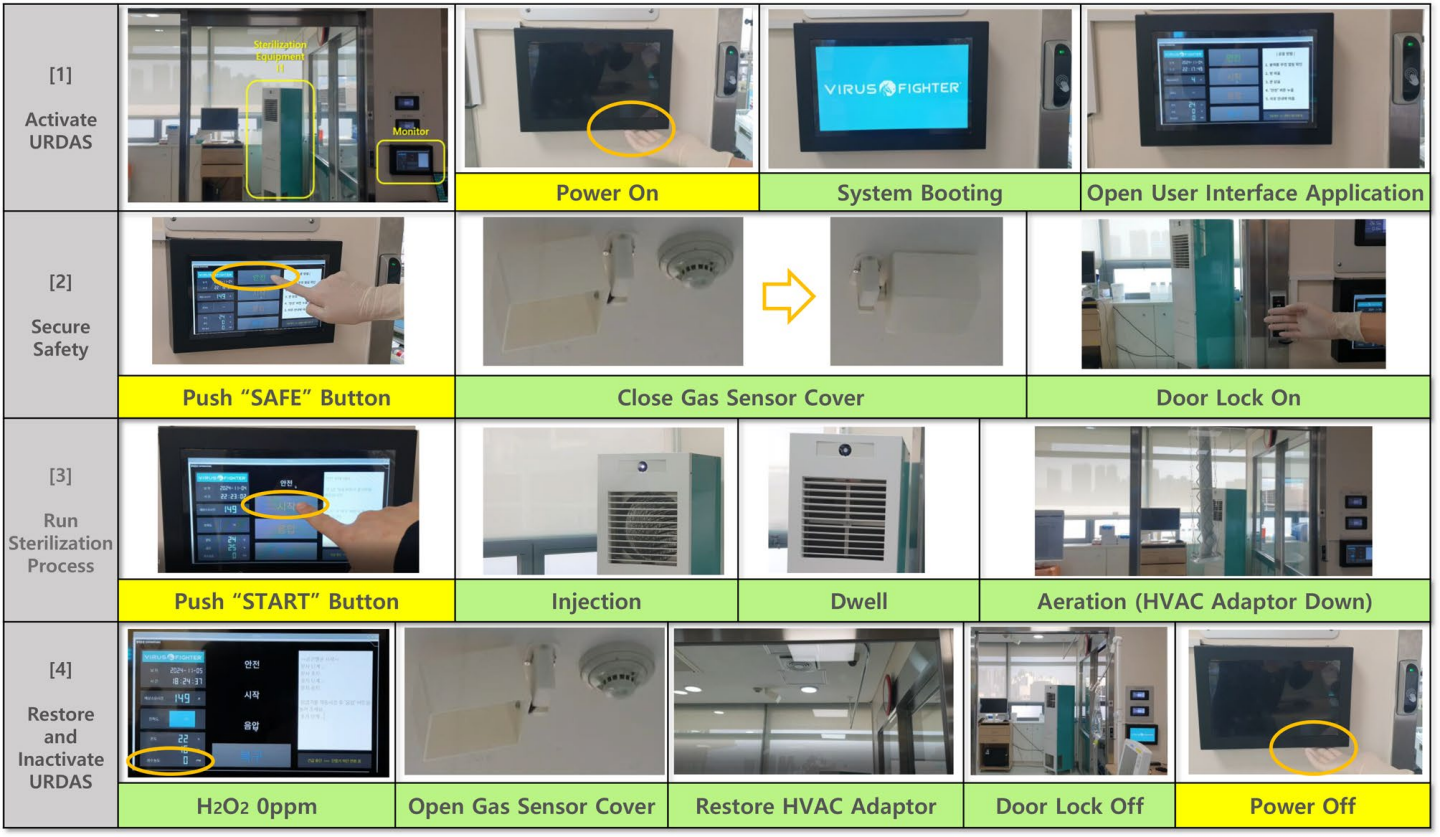
Operation process of an “untact” room disinfection automation system using dry-fogged hydrogen peroxide

The URDAS includes a human interface monitor and a main controller outside the room as part of the automated system, along with a room disinfection equipment unit, human detection sensor, gas sensor cover, HVAC adaptor, door sensor, and safety lock (Fig. 2). The safety system includes human-detection sensors, door sensors, and safety locks. Upon activation, all the room doors were automatically locked to prevent entry. After disinfection was completed, the operator verified that the hydrogen peroxide concentration on the human interface monitor reached 0 ppm. Pressing the termination button releases safety locks and restores room accessibility.

The room-temperature disinfection process using hydrogen peroxide was conducted in three distinct stages (Fig. 2). First, during the fogging and injection phase, the hydrogen peroxide solution is atomized into dry-fog (< 10 μm diameter) and dispersed to saturate the target surfaces in a single room. Second, during the dwelling phase, the dHP is allowed to form micro-condensations on the surfaces and in the air. Reactive oxygen species generated from the decomposition of hydrogen peroxide interact with microorganisms and destroy microbial cellular components. Finally, during the aeration phase, all residual hydrogen peroxide is rapidly eliminated from the disinfected environment via a catalytic filter and a high-efficiency particulate air filter connected to the hospital’s HVAC system. Although the core disinfection process is automated, manual cleaning is necessary to remove surface stains contaminated with organic matter.

Our operation setting consisted of a 6-minute fogging and injection phase, 20-minute dwelling phase, and 100-minute aeration phase. To ensure effective dHP dispersion throughout the room, the device was positioned at a 45-degree diagonal angle from the entrance.

### Statistical analysis

The proportion of sites from which pathogens were recovered before and after URDAS application using dHP was compared using Fisher’s exact test. The total number of pathogen-positive samples obtained from single isolation rooms before and after URDAS application using dHP was compared using the Yates’ corrected chi-square test. Statistical analyses were performed using SPSS software (version 23.0; SPSS Inc., Chicago, IL, USA). Two-sided *P* values < 0.05 were considered significant.

## Results

### Distribution of multidrug-resistant microorganisms isolated from environmental cultures

Between January and March 2023, 26 patients were hospitalized in 19 isolation rooms for vancomycin-resistant enterococci (VRE; *n* = 11), carbapenem-resistant *Enterobacterales* spp. (CRE; *n* = 7), coronavirus disease-2019 (*n* = 6), and tuberculosis (*n* = 2). During the study period, 387 swab samples were collected to identify the contaminated sites in single isolation rooms. Seventy-four MDROs were identified, including VRE (*n* = 42; 56.8%), carbapenem-resistant *Acinetobacter baumannii* (*n* = 14; 18.9%), CRE (*n* = 10; 13.5%), and methicillin-resistant *Staphylococcus aureus* (*n* = 8; 10.8%). The highest positive rate of environmental cultures around patients was observed in those with VRE (22.7%), followed by those with CRE (19.4%), coronavirus disease-2019 (15.5%), and tuberculosis (7.8%). Among the patients with VRE, VRE accounted for the highest proportion of isolated pathogens (19.3%). Among patients with CRE, VRE was the most frequently isolated pathogen (16.0%), followed by CRE (12.0%). MDROs were most often detected on bedsheets (*n* = 17, 22.8%), followed by bed rails (*n* = 5, 6.8%), bed remote controllers (*n* = 5, 6.8%), and removable bed tables (*n* = 4, 5.4%). Interestingly, all types of MDROs were often detected on bedsheets near the patient’s head and legs. VRE, the most frequently isolated MDRO, was detected primarily in bedside rails (*n* = 4, 9.5%), bed-removable tables (*n* = 2, 4.8%), infusion pumps (*n* = 1, 2.4%), and pole stands (*n* = 1, 2.4%; Table 1).

**Table 1:** Distribution of multidrug-resistant microorganisms isolated from environmental swab cultures from hospital rooms

	Total number of trials	No. of positive cultures	VRE	CRAB	CRE	MRSA	Total
Sites, *n* (%)							
Bed-removable table	26	15 (57.7)	2 (4.8)	0	0	2 (25)	4 (5.4)
Infusion pump and pole stand	26	7 (26.9)	1 (2.4)	0	0	0	1 (1.4)
Bedside shelves	26	14 (53.8)	0	0	1 (10)	0	1 (1.4)
Bedside rails	26	18 (69.2)	4 (9.5)	0	1 (10)	0	5 (6.8)
Bed remote controller	26	20 (76.9)	3 (7.1)	0	1 (10)	1 (12.5)	5 (6.8)
Bedsheets (head)	26	20 (76.9)	3 (7.1)	1 (7.1)	1 (10)	2 (25)	7 (9.5)
Bedsheets (leg)	26	21 (80.8)	7 (16.7)	1 (7.1)	1 (10)	1 (12.5)	10 (13.5)
Ventilator and infusion line	10	3 (30.0)	0	0	0	0	0
Patients’ curtain	19	4 (21.1)	0	0	0	0	0
Patients’ monitor	5	1 (20.0)	1 (2.4)	0	0	0	1 (1.4)
Pole stand	5	3 (60.0)	1 (2.4)	0	0	0	1 (1.4)
Blood pressure cuff	15	8 (53.3)	0	2 (14.3)	0	0	2 (2.7)
Oxygen generator and suction	11	7 (63.6)	1 (2.4)	3 (21.4)	0	0	4 (5.4)
Room corners	68	61 (89.7)	14 (33.3)	6 (42.9)	3 (30)	1 (12.5)	24 (32.4)
Washbasin body	14	13 (92.6)	1 (2.4)	0	0	0	1 (1.4)
Washbasin faucet	14	9 (64.3)	1 (2.4)	0	0	0	1 (1.4)
Toilet handle	12	9 (64.3)	0	0	2 (20)	0	2 (2.7)
Toilet seats	12	7 (58.3)	1 (2.4)	0	0	0	1 (1.4)
Door handles	12	6 (50.0)	0	0	0	1 (12.5)	1 (1.4)
Around anteroom entrance	5	4 (80.0)	1 (2.4)	0	0	0	1 (1.4)
Portable negative pressure machines	3	3 (100)	1 (2.4)	1 (7.1)	0	0	2 (2.7)
**Reason for patient isolation**							
VRE (*n* = 11)	144	88 (61.1)	17 (40.5)	0	0	4 (50)	21 (28.4)
COVID-19 (*n* = 6)	95	60 (63.2)	12 (28.6)	9 (64.3)	1 (10)	0	22 (29.7)
Tuberculosis (*n* = 2)	34	30 (88.2)	1 (2.4)	0	0	0	1 (1.4)
CRE (*n* = 7)	114	75 (65.8)	12 (28.6)	5 (35.7)	9 (90)	4 (50)	30 (40.5)
Total isolated, *n* (%)	387	253 (65.4)	42 (56.8)	14 (18.9)	10 (13.5)	8 (10.8)	74

Abbreviations: COVID-19, coronavirus disease 2019; CRAB, carbapenem-resistant *Acinetobacter baumannii*; CRE, carbapenem-resistant *Enterobacterales*; MRSA, methicillin-resistant *Staphylococcus aureus*; VRE, vancomycin-resistant enterococci

### Efficacy of URDAS using dHP for terminal room decontamination

Environmental cultures from inanimate surfaces and room air were obtained before and after disinfection using the URDAS in a single isolation room. Six terminal decontamination cycles were performed in an identical single isolation room equipped with URDAS, each following the discharge of the patient who had occupied the room. During the terminal room decontamination cycles with URDAS, all 6log_10_ biologic indicators containing *G. stearothermophilus* were inactivated. No microorganisms were recovered from the sampled surfaces after the second and third decontamination cycles (Table 2). Overall, 29 microorganisms were detected in 78 samples (37.2%) before decontamination, compared to only 7 microorganisms in 78 samples (9.0%) after decontamination (*P* < 0.01). Even after surface and space disinfection, the most frequently isolated bacteria were the coagulase-negative and *Enterococcus* species (Table 2). The removable tables and bedsheets used by patients often remained contaminated even after disinfection (Table 3). In the fifth case, *Enterococcus* species were isolated from a single isolation room occupied by a patient colonized with VRE.

**Table 2:** Distribution of microorganisms isolated from environmental swab cultures in a single isolation room before and after decontamination with dry-fogged hydrogen peroxide system

	1st		2nd		3rd		4th		5th		6th		Total		
	PRE	POST	PRE	POST	PRE	POST	PRE	POST	PRE	POST	PRE	POST	PRE	POST	*p*-value
Reasons for patient isolation	Trauma (CRAB)		Post-operative deconditioning		Trauma (VRE)		Cerebral aneurysm coil embolization		Microvascular decompression (brain)		Percutaneous coronary intervention				
**Sites,** ***n*** **(%)**															
Bed-removable table	-	-	-	-	A, D	-	-	-	A	H, I	-	A, F	A, D	A, H,I, F	
Infusion pump and pole stand	-	-	-	-	-	-	-	-	-	-	-	-	-	-	
Bedside shelves	-	-	-	-	-	-	-	-	-	-	-	-	-	-	
Bedside rails	A, C	-	-	-	A	-	-	-	-	-	-	-	A, C	-	
Bed remote controller	-	-	-	-	B	-	-	-	-	-	-	-	B	-	
Bedsheets (head)	-	A, C	-	-	A, B	-	-	-	A	-	-	-	A, B	A, C	
Bedsheets (leg)	-	-	-	-	A, C	-	A, G	D	B	-	A, G	-	A, B,C, G	D	
Ventilator and infusion line	-	-	-	-	A, B,D	-	-	-	-	-	A, J	-	A, B,D, J	-	
Patients’ curtain	-	-	-	-	-	-	-	-	-	-	-	-	-	-	
Patients’ monitor	A, C	-	-	-	A, B,K	-	-	-	-	-	-	-	A, B,C, K	-	
Pole stand	-	-	-	-	A, B	-	-	-	-	-	-	-	A, B	-	
Blood pressure cuff	-	-	-	-	-	-	-	-	-	-	-	-	-	-	
Oxygen generator and suction bottle	-	-	-	-	-	-	-	-	-	-	-	-	-	-	
**Total isolated**	4	2	0	0	16	0	2	1	3	2	4	2	29	7	< 0.01

Abbreviations: CRAB, carbapenem-resistant *Acinetobacter baumannii*; VRE, vancomycin-resistant enterococci

(A) *Staphylococcus epidermidis*, (B) *Staphylococcus hominis*, (C) *Staphylococcus haemolyticus*, (D) *Staphylococcus capitis*, (E) *Staphylococcus caprae*, (F) *Staphylococcus cohnii*, (G) *Corynebacterium tuberculostearicum*, (H) *Enterococcus faecium* (no VRE), (I) *Enterococcus faecalis* (non-VRE), (J) *Corynebacterium bovis*, and K. *Dermabacter hominis*

**Table 3:** Distribution of microorganisms isolated from environmental air cultures in a single isolation room before and after decontamination with dry-fogged hydrogen peroxide system

	Bacteria	Fungus
**1st round of disinfection**		
Pre-culture	*Brevibacterium casei* *Staphylococcus haemolyticus* *Micrococcus luteus* *Bacillus licheniformis* *Paenibacillus barengoltzii* *Staphylococcus hominis* *Micrococcus spp.*	*Alternaria alternata*
Post-culture	*Moraxella osloensis* *Kocuria marina* *Staphylococcus xylosus*	No growth
**2nd round of disinfection**		
Pre-culture	*Bacillus cereus* *Moraxella osloensis* *Enterococcus faecium* *Micrococcus luteus* *Staphylococcus haemolyticus* *Serratia marcescens* *Kocuria rhizophila*	*Trichophyton terrestre*
Post-culture	*Staphylococcus hominis*	*Trichophyton mentagrophytes*
**3rd round of disinfection**		
Pre-culture	*Staphyloccus epidermidis* *Staphylococcus hominis* *Staphylococcus haemolytus* *Bacillus pumilus*	*Trichophyton tonsurans*
Post-culture	*Staphylococcus hominis* *Moraxella osloensis* *Micrococcus luteus*	*Trichophyton rubrum*
**4th** **round of disinfection**		
Pre-culture	*Micrococcus luteus* *Staphylococcus aureus* *Paracoccus yeei* *Staphylococcus caprae*	*Aspergillus versicolor*
Post-culture	*Micrococcus luteus* *Staphylococcus hominis* *Dermabacter hominis*	*Aspergillus flavus* *Aspergillus versicolor*
**5th round of disinfection**		
Pre-culture	*Corynebacterium mucifaciens* *Bacillus pumilus* *Staphylococcus hominis* *Metabacillus idriensis*	*Aspergillus versicolor* *Penicillium capsulatum*
Post-culture	*Micrococcus luteus* *Staphylococcus hominis*	*Aspergillus versicolor*
**6th round of disinfection**		
Pre-culture	*Micrococcus luteus* *Staphylococcus warneri* *Dermacoccus nishinomiyaensis*	*Penicillium sp.*
Post-culture	*Micrococcus luteus* *Priestia megaterium* *Bacillus licheniformis*	*Aspergillus flavus* *Cladosporium cladosporioides* *Aspergillus fumigatus*

### Air culture results before and after terminal room decontamination using the URDAS with dHP

Air cultures were also performed before and after disinfection using the URDAS in a single isolation room (Table 3). Thirty-six species were isolated from the air cultures before disinfection, which decreased to 23 species after disinfection, representing a 36.1% reduction (Supplementary Table S4). However, all 12 samples (100%) were positive before decontamination, and 11 of the 12 (91.7%) remained positive after decontamination (*P* = 0.537). Maintaining sterile room air proved to be challenging, although the number of bacterial species isolated after disinfection tended to decrease (Table 3). In the fourth and subsequent cases, *Aspergillus* spp. were isolated even after disinfection.

## Discussion

Our findings showed that high-touch surfaces near patients can remain contaminated with nosocomial pathogens in clinical settings, particularly when occupied by patients with MDROs. To improve the medical environment, a novel URDAS using dHP achieved intensive disinfection of inanimate surfaces within a relatively short running time. Despite their limitations, the challenge lies in optimizing the value of new technologies in dynamic and demanding medical settings where MDROs are prevalent.

Our findings align with prior research showing that multidrug-resistant gram-positive and gram-negative bacteria contaminate hospital environments and facilities [15, 16]. Among MDROs, VRE is most often found on high-touch surfaces near patients, underscoring the critical role these surfaces play as reservoirs for VRE transmission in healthcare settings [17, 18]. The same was true for CRE and carbapenem-resistant *Acinetobacter baumannii*, which were isolated from bedsheets, bedrails, monitors, ventilators, sinks, electromanometers, keyboards, and infusion pumps [19, 20]. Bedsheets were the most frequently contaminated site in this study, which is consistent with previous studies [21, 22].

In this study, hydrogen peroxide, an oxidizing agent that produces highly reactive hydroxyl radicals, proved to be effective in attacking essential cellular components. Two major hydrogen peroxide systems are available: vaporized H_2_O_2_ systems, which deliver heat-generated vapors of 30% weight/weight aqueous H_2_O_2_ through a high-velocity air system, and aerosolized H_2_O_2_ systems, which deliver pressure- or ultrasonic-generated aerosols of 5% weight/weight aqueous H_2_O_2_ through a unidirectional nozzle [6]. In contrast to conventional vaporized or aerosolized H_2_O_2_ systems, fogging (fumigation) with dHP is an advanced disinfection technology. dHP uses compressed air to atomize the hydrogen peroxide solution into ultra-fine droplets (typically < 10 μm in diameter), producing a dry mist that behaves more like a gas. This allowed the disinfectant to fill the entire space rapidly and evenly, including hard-to-reach and shadowed areas, without causing surface condensation. Consequently, dHP offers a particularly effective and practical alternative for comprehensive spatial disinfection in healthcare environments, as demonstrated in our study [23–25].

The URDAS was designed to be user-friendly, allowing most medical staff to operate it remotely rather than requiring skilled experts. Furthermore, as a no-touch decontamination system, it circumvents the issues associated with manual disinfection. It is equipped with additional safety features to minimize operational errors, including an HVAC adaptor, sensor devices, locking mechanisms, and a control terminal (Fig. 1). In our study, the URDAS operated for an approximate running time of 2.5 h, with ongoing optimization efforts focused on reducing the aeration time. However, it should be used only in vacated rooms, and as a supplement to manual cleaning, as biological soiling on hospital surfaces can reduce the effectiveness of disinfection.

Consistent with our findings, previous studies have demonstrated that hydrogen peroxide systems effectively reduce MDRO contamination on hospital surfaces [6, 26, 27]. Nevertheless, our study showed that even after URDAS-based disinfection, coagulase-negative bacteria were often isolated from inanimate surfaces in single-isolation rooms. Studies have identified decreased susceptibility of catalase-positive bacteria to hydrogen peroxide, suggesting that catalase can degrade hydrogen peroxide [28, 29]. The resistance of microorganisms to hydrogen peroxide largely depends on the production of superoxide dismutases (SODs), catalase, and other peroxidases. Conversely, another conflicting study suggested otherwise, highlighting the need for further in-depth research to optimize hydrogen peroxide disinfection protocols [30].

Our study also revealed a relatively low efficacy against fungi in air culture tests. This aligns with prior research indicating that fungal spores, such as *Aspergillus* species, may exhibit higher resistance to hydrogen peroxide owing to their structural characteristics [31, 32]. For instance, studies have noted that, while bacterial pathogens are effectively reduced by hydrogen peroxide systems, fungal spores often require high concentrations or prolonged exposure times for complete eradication [10, 32]. These findings suggest the need for further optimization of hydrogen peroxide protocols, such as adjusting the concentration levels or combining hydrogen peroxide with other disinfectants, to enhance antifungal efficacy.

Unfortunately, bedsheets often remained contaminated in our study even after disinfection using URDAS, similar to previous findings [33]. Most studies have reported the efficacy of hydrogen peroxide against microorganisms dried on hard, non-porous surfaces; however, data on its effectiveness against pathogens on porous surfaces such as textiles or cotton are limited [6, 10, 26, 27]. Therefore, terminal room disinfection should include the removal of used bedsheets, curtains, and disposable items in addition to non-touch automated disinfection following routine cleaning. However, hydrogen peroxide appears to help reduce the total pathogen load on fabric materials, such as curtains [34, 35].

Our findings also suggest the possibility of rapid recontamination after disinfection with URDAS as soon as patients and healthcare staff are readmitted, which is consistent with a previous study [36]. Several pathogens that were not identified before disinfection were often isolated from surfaces or air cultures obtained in our study, even after disinfection. No studies have documented the long-term effects of hydrogen peroxide disinfection on recontamination rates, bacterial transmission, or fungal inflow from the outside atmosphere. Moreover, the optimal frequency of application for achieving infection control goals has not yet been established.

Our study design intentionally omitted manual cleaning prior to dHP application to directly assess the standalone effectiveness of automated touchless disinfection. This approach reflects real-world situations where entering a contaminated space may pose significant risks to healthcare staff, such as during outbreaks of highly contagious or lethal pathogens. Our findings suggest that the dHP can serve as an auxiliary intervention for terminal room disinfection in situations where manual cleaning is impractical or poses a significant risk to personnel.

Our study had several limitations. First, it lacked a direct comparison of the performance between URDAS using dHP and routine manual cleaning. However, this limitation might be partially offset by the well-documented inefficiencies of manual cleaning, including persistent contamination (5–30% of surfaces) and operator-dependent failures, as described in prior studies [37, 38]. Thus, URDAS was designed to overcome these systemic issues through automation, consistency, and reduced human critical errors in high-MDRO settings. Future controlled studies should quantify the efficacy of URDAS using dHP compared with manual cleaning. Second, due to the small sample size, our findings may not be generalizable to other patient populations. However, sufficient swab cultures were obtained from various hospital environments. Third, environmental cultures collected prior to URDAS application were obtained before routine terminal manual cleaning and disinfection. Therefore, our findings did not allow for an evaluation of the effectiveness of routine terminal cleaning and disinfection. Fourth, we were unable to quantify airborne microbial contamination, such as CFU/m³ or assess the degree of microbial load reduction. Future studies should incorporate quantitative colony counting for a more comprehensive assessment of airborne microbial contamination. Fifth, when performing air cultures after URDAS disinfection, it is difficult to maintain a completely closed space to operate the air-culture device. Thus, the possibility of re-contamination due to external factors could not be ruled out after disinfection was completed. Further studies with improved research designs are required to address these issues.

## Conclusions

Our findings provide foundational evidence for the importance of thorough environmental cleaning after patient discharge. This study demonstrated that URDAS using dHP is an effective adjunctive infection control measure for terminal room decontamination, achieving significant reductions in surface microbial contamination and a partial reduction in airborne microbial species in single isolation rooms. The adoption of automated, touchless disinfection technologies such as URDAS represents a paradigm shift in hospital space disinfection practices. Overcoming material- and pathogen-specific disinfection challenges through optimization processes will further enhance the value of innovative disinfection systems in real-world healthcare settings.

## Electronic supplementary material

Below is the link to the electronic supplementary material.

Supplementary Material 1

## Data Availability

No datasets were generated or analysed during the current study.

## Abbreviations

CRE: carbapenem-resistant *Enterobacterales *spp
dHP: dry-fogged hydrogen peroxide
HVAC: heating, ventilation, and air conditioning
MDROs: multidrug resistance organisms
URDAS: “untact” room disinfection automation system
VRE: vancomycin-resistant enterococci
