# Supplementary material for: Assessment of dry-fogged hydrogen peroxide as an “untact” room disinfection automation system for rapid terminal decontamination of a single isolation room in a healthcare institution
Source: Antimicrob Resist Infect Control. 2025 Jul 26;14:92. doi: 10.1186/s13756-025-01613-7 (PMC12296622; doi:10.1186/s13756-025-01613-7)
Supplement: Supplementary file 1 — Supplementary Material 1 [file 13756_2025_1613_MOESM1_ESM.docx]

**Supplementary Table S1.** Distribution of pathogens isolated from environmental swab cultures of patient surroundings.

| **Pathogens, *n* (%)** | **A** | **B** | **C** | **D** | **E** | **F** | **G** | **H** | **I** | **J** | **K** | **L** | **M** | **Total** |
| --- | --- | --- | --- | --- | --- | --- | --- | --- | --- | --- | --- | --- | --- | --- |
| CRE | 0 | 0 | 2 (7.4) | 1 (4.3) | 1 (2.6) | 2 (5.6) | 1 (2.4) | 0 | 0 | 0 | 0 | 0 | 0 | 6 (2.5) |
| *K. pneumoniae* (non-CRE) | 1 (4.2) | 0 | 0 | 0 | 0 | 0 | 0 | 0 | 0 | 0 | 0 | 0 | 0 | 1 (0.4) |
| *Staphylococcus* spp. |  |  |  |  |  |  |  |  |  |  |  |  |  |  |
| MRSA | 2 (8.3) | 0 | 0 | 0 | 1 (2.6) | 2 (5.6) | 1 (2.4) | 0 | 0 | 0 | 0 | 0 | 0 | 6 (2.5) |
| *Staphylococcus* CoN^a^ | 7 (29.2) | 3 (27.3) | 12 (44.4) | 11 (47.8) | 20 (52.6) | 19 (52.8) | 22 (53.7) | 4 (80) | 3 (60) | 0 | 0 | 6 (37.5) | 5 (38.5) | 112 (46.1) |
| *Corynebacterium* spp. | 1 (4.2) | 1 (9.1) | 4 (14.8) | 0 | 3 (7.9) | 4 (11.1) | 3 (7.3) | 0 | 1 (20) | 0 | 1 (33.3) | 3 (18.8) | 1 (7.7) | 22 (9.1) |
| *Acinetobacter* spp. |  |  |  |  |  |  |  |  |  |  |  |  |  |  |
| Non-CRAB | 0 | 0 | 2 (7.4) | 1 (4.3) | 0 | 1 (2.8) | 0 | 0 | 0 | 0 | 0 | 0 | 0 | 4 (1.6) |
| CRAB | 0 | 0 | 0 | 0 | 0 | 1 (2.8) | 1 (2.4) | 0 | 0 | 0 | 0 | 2 (12.5) | 3 (23.1) | 7 (2.9) |
| Non-*baumannii* | 0 | 0 | 0 | 0 | 0 | 0 | 0 | 0 | 0 | 0 | 0 | 1 (6.25) | 0 | 1 (0.4) |
| VRE | 2 (8.3) | 1 (9.1) | 0 | 4 (17.4) | 3 (7.9) | 3 (8.3) | 7 (17.1) | 0 | 0 | 1 (100) | 1 (33.3) | 0 | 1 (7.7) | 23 (9.5) |
| *E. faecalis* (non-VRE) | 2 (8.3) | 1 (9.1) | 2 (7.4) | 1 (4.3) | 3 (7.9) | 0 | 1 (2.4) | 0 | 0 | 0 | 0 | 0 | 0 | 10 (4.1) |
| *E. faecium* (non-VRE) | 1 (4.2) | 1 (9.1) | 1 (3.7) | 1 (4.3) | 3 (7.9) | 1 (2.8) | 2 | 1 (20) | 0 | 0 | 1 (33.3) | 0 | 0 | 12 (4.9) |
| *Chryseobacterium* spp. | 1 (4.2) | 0 | 1 (3.7) | 0 | 0 | 0 | 0 | 0 | 0 | 0 | 0 | 1 (6.25) | 0 | 3 (1.2) |
| *Leuconostoc* spp. | 2 (8.3) | 0 | 0 | 1 (4.3) | 1 (2.6) | 0 | 0 | 0 | 0 | 0 | 0 | 0 | 0 | 4 (1.6) |
| *Moraxella* spp. | 0 | 0 | 1 (3.7) | 0 | 0 | 0 | 0 | 0 | 0 | 0 | 0 | 0 | 0 | 1 (0.4) |
| *Kocuria* spp. | 0 | 0 | 1 (3.7) | 0 | 0 | 0 | 0 | 0 | 0 | 0 | 0 | 0 | 0 | 1 (0.4) |
| Micrococcus luteus | 1 (4.2) | 1 (9.1) | 0 | 1 (4.3) | 1 (2.6) | 0 | 1 (2.4) | 0 | 1 (20) | 0 | 0 | 1 (6.25) | 2 (15.4) | 9 (3.7) |
| *Streptococcu*s spp. | 1 (4.2) | 0 | 0 | 1 (4.3) | 0 | 1 (2.8) | 0 | 0 | 0 | 0 | 0 | 0 | 0 | 3 (1.2) |
| *Bacillus* spp. | 2 (8.3) | 1 (9.1) | 1 (3.7) | 0 | 2 (5.3) | 1 (2.8) | 0 | 0 | 0 | 0 | 0 | 1 (6.25) | 1 (7.7) | 9 (3.7) |
| *Facklamia hominis* | 0 | 1 (9.1) | 0 | 0 | 0 | 0 | 0 | 0 | 0 | 0 | 0 | 0 | 0 | 1 (0.4) |
| *Brevibacterium* spp. | 0 | 0 | 0 | 0 | 0 | 1 (2.8) | 0 | 0 | 0 | 0 | 0 | 0 | 0 | 1 (0.4) |
| *Pedicoccus pentosaceus* | 0 | 1 (9.1) | 0 | 0 | 0 | 0 | 0 | 0 | 0 | 0 | 0 | 0 | 0 | 1 (0.4) |
| *Brevundimonas* spp. | 1 (4.2) | 0 | 0 | 0 | 0 | 0 | 0 | 0 | 0 | 0 | 0 | 0 | 0 | 1 (0.4) |
| *Rothia amarae* | 0 | 0 | 0 | 0 | 0 | 0 | 1 (2.4) | 0 | 0 | 0 | 0 | 0 | 0 | 1 (0.4) |
| *Lactobacillus* spp. | 0 | 0 | 0 | 0 | 0 | 0 | 1 (2.4) | 0 | 0 | 0 | 0 | 0 | 0 | 1 (0.4) |
| *Methylobacterium* spp. | 0 | 0 | 0 | 0 | 0 | 0 | 0 | 0 | 0 | 0 | 0 | 1 (6.25) | 0 | 1 (0.4) |
| *Lactococcus lactis* | 0 | 0 | 0 | 1 (4.3) | 0 | 0 | 0 | 0 | 0 | 0 | 0 | 0 | 0 | 1 (0.4) |
| Total isolated | 24 | 11 | 27 | 23 | 38 | 36 | 41 | 5 | 5 | 1 | 3 | 16 | 13 | 243 |

A: bed-removable table; B: infusion pump and pole stand; C: bedside shelves; CoN: coagulase-negative; CRAB: carbapenem-resistant *Acinetobacter baumannii*; CRE: carbapenem-resistant *Enterobacterales* spp.; D: bedside rails; E: bed-remote controller; F: bedsheets (head); G: bedsheets (leg); H: ventilator and infusion line; I: patient curtain; J: patient monitor; K: pole stand; L: blood pressure cuff; M: oxygen generator or suction bottle; VRE: vancomycin-resistant enterococci.

^a^ *Staphylococcus* CoN includes *S. haemolyticus*, *caprae*, *capitis*, *hominis*, *epidermidis*, *pettenkoferi*, and *warneri*.

**Supplementary Table S2.** Distribution of pathogens isolated from environmental swab cultures from hospital facilities.

| **Pathogens, *n* (%)** | **N** | **O** | **P** | **Q** | **R** | **S** | **T** | **U** | **Total** |
| --- | --- | --- | --- | --- | --- | --- | --- | --- | --- |
| CRE | 3 (2.2) | 0 | 0 | 2 (11.8) | 0 | 0 | 0 | 0 | 5 (2.0) |
| *Klebsiella oxytoca* (non-CRE) | 0 | 1 (3.3) | 0 | 0 | 0 | 0 | 0 | 0 | 1 (0.4) |
| *Staphylococcus* spp. |  |  |  |  |  |  |  |  |  |
| MRSA | 1 (0.7) | 0 | 0 | 0 | 0 | 1 (9.0) | 0 | 0 | 2 (0.8) |
| *Staphylococcus* CoN^a^ | 46 (33.3) | 5 (16.7) | 3 (15) | 2 (11.8) | 10 (66.7) | 4 (36.4) | 4 (44.4) | 4 (50.0) | 78 (31.5) |
| *Corynebacterium* spp. | 10 (7.2) | 1 (3.3) | 1 (5) | 0 | 2 (13.3) | 1 (9.0) | 1 (11.1) | 0 | 16 (6.5) |
| *Acinetobacter* spp. |  |  |  |  |  |  |  |  |  |
| Non-CRAB | 0 | 3 (10) | 3 (15) | 2 (11.8) | 0 | 1 (9.0) | 0 | 0 | 9 (3.6) |
| CRAB | 6 (4.3) | 0 | 0 | 0 | 0 | 0 | 0 | 1 (12.5) | 7 (2.8) |
| Non-baumannii | 6 (4.3) | 0 | 0 | 0 | 0 | 1 (9.0) | 0 | 0 | 7 (2.8) |
| Enterococcus spp.^b^ | 2 (1.4) | 0 | 0 | 0 | 1 (6.7) | 0 | 0 | 0 | 3 (1.2) |
| VRE | 14 (10.1) | 1 (3.3) | 1 (5) | 0 | 1 (6.7) | 0 | 1 (11.1) | 1 (12.5) | 19 (7.7) |
| *E. faecalis* (non-VRE) | 4 (2.9) | 2 (6.7) | 0 | 3 (17.6) | 0 | 0 | 0 | 0 | 9 (3.6) |
| *E. faecium* (non-VRE) | 5 (3.6) | 0 | 0 | 1 (5.9) | 0 | 0 | 0 | 0 | 6 (2.4) |
| *Chryseobacterium* spp. | 7 (5.1) | 0 | 1 (5) | 1 (5.9) | 0 | 0 | 0 | 0 | 9 (3.6) |
| *Leuconostoc* spp. | 0 | 0 | 0 | 1 (5.9) | 0 | 1 (9.0) | 0 | 0 | 2 (0.8) |
| *Moraxella* spp. | 2 (1.4) | 1 (3.3) | 2 (10) | 2 (11.8) | 0 | 0 | 0 | 0 | 7 (2.8) |
| *Kocuria* spp. | 0 | 1 (3.3) | 0 | 0 | 0 | 0 | 0 | 0 | 1 (0.4) |
| *Micrococcus luteus* | 5 (3.6) | 1 (3.3) | 0 | 0 | 0 | 0 | 1 (11.1) | 0 | 7 (2.8) |
| *Streptococcus* spp. | 0 | 1 (3.3) | 2 (10) | 0 | 0 | 0 | 0 | 0 | 3 (1.2) |
| *Bacillus* spp. | 11 (8.0) | 5 (16.7) | 3 (15) | 2 (11.8) | 1 (6.7) | 1 (9.0) | 1 (11.1) | 1 (12.5) | 25 (10.1) |
| *Brevibacterium* spp. | 0 | 1 (3.3) | 1 (5) | 0 | 0 | 0 | 0 | 0 | 2 (0.8) |
| Brevundimonas spp. | 3 (2.2) | 0 | 0 | 0 | 0 | 0 | 0 | 0 | 3 (1.2) |
| *Enterobacter* spp. | 0 | 2 (6.7) | 1 (5) | 0 | 0 | 0 | 0 | 0 | 3 (1.2) |
| *Peudomonas* spp. | 6 (4.3) | 1 (3.3) | 1 (5) | 0 | 0 | 0 | 0 | 0 | 8 (3.2) |
| *Stenotrophomonas maltophilia* | 1 (0.7) | 0 | 0 | 0 | 0 | 0 | 0 | 0 | 1 (0.4) |
| *Neisseria flavescens* | 0 | 1 (3.3) | 0 | 0 | 0 | 0 | 0 | 0 | 1 (0.4) |
| *Delftia acidovorans* | 0 | 1 (3.3) | 0 | 0 | 0 | 0 | 0 | 0 | 1 (0.4) |
| *Acidovorax temperans* | 0 | 2 (6.7) | 0 | 0 | 0 | 0 | 0 | 0 | 2 (0.8) |
| *Paenibacillus* spp. | 1 (0.7) | 0 | 0 | 0 | 0 | 0 | 0 | 1 (12.5) | 2 (0.8) |
| *Rhizobium radiobacter* | 1 (0.7) | 0 | 0 | 0 | 0 | 0 | 0 | 0 | 1 (0.4) |
| *Microbacterium* spp. | 2 (1.4) | 0 | 0 | 0 | 0 | 0 | 0 | 0 | 2 (0.8) |
| *Sphingomonas* spp. | 0 | 0 | 0 | 1 (5.9) | 0 | 1 (9.0) | 0 | 0 | 2 (0.8) |
| *Aerococcus viridans* | 0 | 0 | 1 (5) | 0 | 0 | 0 | 0 | 0 | 1 (0.4) |
| *Brevibacillus* spp. | 1 (0.7) | 0 | 0 | 0 | 0 | 0 | 0 | 0 | 1 (0.4) |
| *Niallia* spp. | 0 | 0 | 0 | 0 | 0 | 0 | 1 (11.1) | 0 | 1 (0.4) |
| *Streptomyces olivaceus* | 1 (0.7) | 0 | 0 | 0 | 0 | 0 | 0 | 0 | 1 (0.4) |
| Total isolated | 138 | 30 | 20 | 17 | 15 | 11 | 9 | 8 | 248 |

CoN, coagulase-negative staphylococci; CRAB, carbapenem-resistant *Acinetobacter baumannii*; CRE, carbapenem-resistant *Enterobacterales*; MRSA, methicillin-resistant *Staphylococcus aureus*; N, room corners; O, wash basin body; P, wash basin faucet; Q, toilet handle; R, toilet seats; S, door handles; T, area around the anteroom entrance; U, portable negative pressure machines; VRE, vancomycin-resistant enterococci.

^a^ *Staphylococcus* CoN, including *S. haemolyticus*, *caprae*, *capitis*, *hominis*, *epidermidis*, and *pettenkoferi*.

^b^ *Enterococcus* spp., including *E. Gallinarum* and *E. avium*.

**Supplementary Table S3.** Specifications of an “untact” room disinfection automation system using dry-fogged hydrogen peroxide for rapid terminal room decontamination.

| **Parameter** | **Specification** |
| --- | --- |
| Microbial reduction level | 6Log |
| Space sterilization capacity (m^3^/cycle) | 120–180 |
| Main unit dimensions (length × width × height, mm) | 387 × 542 × 1,750 |
| Operating temperature (℃) / Humidity (%) | 5–45 / ~99 |
| Hydrogen peroxide concentration (%) | 14 |
| Number of spray devices (units) | 1 |
| Average aerozol size (μm) / spray volume (ml) | ~10 / 40–60 |
| Plasma discharge voltage (kV) | 17.5 |
| Airflow aftereffects (m^3^/min) / performance (min/40 m^3^) | 61 / 180 |

**Supplementary Table S4.** Distribution of bacterial and fungal species isolated from air cultures before and after disinfection with the URDAS.

|  |  | Pre-cultures | Post-cultures |
| --- | --- | --- | --- |
| 1^st^ | Bacteria | 7 | 3 |
|  | Fungus | 1 | 0 |
| 2^nd^ | Bacteria | 7 | 1 |
|  | Fungus | 1 | 1 |
| 3^rd^ | Bacteria | 4 | 3 |
|  | Fungus | 1 | 1 |
| 4^th^ | Bacteria | 4 | 3 |
|  | Fungus | 1 | 2 |
| 5^th^ | Bacteria | 4 | 2 |
|  | Fungus | 2 | 1 |
| 6^th^ | Bacteria | 3 | 3 |
|  | Fungus | 1 | 3 |
| Total | Bacteria | 29 | 15 |
|  | Fungus | 7 | 8 |
|  | Total | 36 | 23 |
